# A Stochastic Model for Detecting Overlapping and Hierarchical Community Structure

**DOI:** 10.1371/journal.pone.0119171

**Published:** 2015-03-30

**Authors:** Xiaochun Cao, Xiao Wang, Di Jin, Xiaojie Guo, Xianchao Tang

**Affiliations:** 1 School of Computer Science and Technology, Tianjin University, Tianjin 300072, China; 2 State Key Laboratory of Information Security, Institute of Information Engineering, Chinese Academy of Sciences, Beijing 100093, China; University of Namur, BELGIUM

## Abstract

Community detection is a fundamental problem in the analysis of complex networks. Recently, many researchers have concentrated on the detection of overlapping communities, where a vertex may belong to more than one community. However, most current methods require the number (or the size) of the communities as a priori information, which is usually unavailable in real-world networks. Thus, a practical algorithm should not only find the overlapping community structure, but also automatically determine the number of communities. Furthermore, it is preferable if this method is able to reveal the hierarchical structure of networks as well. In this work, we firstly propose a generative model that employs a nonnegative matrix factorization (NMF) formulization with a *l_2,1_* norm regularization term, balanced by a resolution parameter. The NMF has the nature that provides overlapping community structure by assigning soft membership variables to each vertex; the *l_2,1_* regularization term is a technique of group sparsity which can automatically determine the number of communities by penalizing too many nonempty communities; and hence the resolution parameter enables us to explore the hierarchical structure of networks. Thereafter, we derive the multiplicative update rule to learn the model parameters, and offer the proof of its correctness. Finally, we test our approach on a variety of synthetic and real-world networks, and compare it with some state-of-the-art algorithms. The results validate the superior performance of our new method.

## Introduction

Many real-world systems can be represented as complex networks, where the vertices represent the components of the systems and the links represent the interactions between them. In complex networks, they share some common properties such as the small-world property [1] and power-law degree distributions [2]. Recently, community structure, one of the most important inherent properties in complex networks, attracts a lot of attentions. It is regarded as groups of vertices with denser connections within groups but sparser connections between them [3]. It is believed that communities play important roles in different real-world systems. It reveals the meaningful topological structure in a wide variety of real-world networks, *e.g.* friendship communities in a social network or protein functional structures in a protein interaction network. Therefore, discovering communities is very crucial for us to understand how different units in a complex system communicate with each other and work together. Furthermore, it provides a valuable insight to the organization and behavior of the network, and offers clues for further investigations. It is thus not surprising that community detection in networks is essential and has been widely investigated over the last few years.

An important problem in community detection is finding overlapping communities, i.e., vertices may belong to more than one community [4]. Overlapping communities exist comprehensively in the real-world networks. For example, in a social network, an individual may participate in several social communities, depending on personal professions, friends, etc. In last decades, a number of approaches for overlapping community detection have been proposed [5–7]. Among these algorithms, stochastic blockmodel, instead of directly detecting communities, is a form of statistical inference for networks and describes how community structures are generated [7]. Here we give a simple example of stochastic blockmodel to formulate community structure. In the blockmodel, we can specify a set of probabilities *p*_*ck*_’s, where *p*_*ck*_ represents the probability of a link between any two vertices in communities *c* and *k*, respectively. Then *p*_*ck*_ can be specified a large value when *c* = *k*, and a small value otherwise. In the generative process, we can create a network that has many links within communities and few between them. We fit the model to the observed network, and then its community structure can be inferred by parameters learning. Thus, stochastic blockmodel seems to be theoretically solid by using statistical inference. Besides, the model generates the expected network similar with the original network, which can help us better understand the structure of the network, and hence it often gets some interpretable and more trustworthy results. Because of the above property, if the model fits a network well, we can further apply it to predict the missing links from the viewpoint of generating the network. More widely, this property also means that the model is not limited to detecting traditional community structure (i.e., a set of communities with dense internal connections and sparse external ones), but any type of community structures (such as hierarchical, bipartite, or k-partite structures and many others [8]), which can be formulated as a model [7]. Therefore, stochastic model has been becoming a type of promising method for overlapping community detection. Specifically, this method requires to explicitly model the network and then infers the community memberships of vertices. Along this line, some recent methods are based on blockmodel or its variations, and employ nonnegative matrix factorization (NMF) to learn their models [9–14]. For example, Zarei *et al.* [9] introduced a vertex-vertex correlation matrix to represent the relationship between vertices, instead of adjacency matrix. Then they applied NMF to analysis this feature matrix and got the overlapping communities. Psorakis *et al.* [10] generated the expectation network by using two nonnegative matrices. Then they utilized a Bayesian NMF which combines the Kullback-Leibler (KL) divergence with the prior model on the two nonnegative matrices to infer overlapping communities from a network, while determining the number of communities. Wang *et al.* [11] proposed three NMF techniques (Symmetric NMF, Asymmetric NMF, and Joint NMF) to detect communities in undirected, directed, and compound networks, respectively. Zhang *et al.* [12] modeled the expectation network by two nonnegative matrices in three factors form. In this way, they could learn the community membership of each vertex as well as the interaction among communities. Zhang *et al.* [13] developed a symmetric binary matrix factorization model to identify overlapping communities. Besides, they could distinguish outliers from overlapping vertices. Cao *et al.* [14] proposed a model consisting of the centrality matrix of vertices and degree matrix of communities. Then based on NMF, they inferred the two types of parameters to identify overlapping communities, hubs, and outliers simultaneously.

However, for a model-based method, one often has to solve the model selection problem, *i.e.* inferring the correct number of communities in a network automatically. In some cases, one can obtain the number of communities in advance. But in most situations, we do not know how many communities in a network because we are often lack of background or domain knowledge. As a result, it is very difficult for us to properly determine the number of communities. Model selection and scalability are two common drawbacks suffered by almost all methods based on stochastic blockmodel. The traditional statistical model selection strategy (e.g. minimum description length [15, 16] or consensus clustering [17]) applied to stochastic models may, in principle, be able to find the number of communities *K* in a consistent and satisfactory manner. But it needs to scan *K* in a large range which makes it too computationally expensive to be applied to real large networks. We find out that, Bayesian model selection [10, 18] uses priors that penalize their model for including too many nonzero parameter values and hence achieves a balance between the number of communities and goodness of fitting the network data, which avoids scanning *K*. However, the priors themselves contain undetermined parameters whose values can influence the number of communities and hence the problem is not completely solved by this approach. Besides, they did not consider the detection of hierarchical structure, which often appears in real networks.

Another key problem in community detection is finding hierarchical communities, where small communities are nested in larger ones. Different resolutions determine the average size of communities [19], which enables us to explore the hierarchical levels of the network. If the resolution is low, the whole network will be divided into several large communities. Extremely, if the resolution is low enough, maybe the whole network will be considered as a largest community. On the contrary, if the resolution is high, the network will be divided into many small communities. Taking the hierarchical structure of a school network as an example, at a low scale, the whole school network can be considered as a community. On the other hand, at a higher scale, each class may be represented as a community. And even each grade can be represented as a community between the two scales. The communities detected at different scales may represent different functional units. Thus, hierarchy can not only show the macroscopical view of the network, but also reveal more detailed community information. Furthermore, as one usually has no knowledge about how large the communities are, so it is necessary to compare the detected communities at different scales.

Recently, some researches have focused on hierarchical community detection. For instance, Blondel *et al.* [20] proposed a heuristic method based on modularity optimization. Furthermore, they merged the small communities if merging these communities increases the modularity. In this way, the hierarchical community structure was built. In [19], Lancichinetti *et al.* introduced a fitness function with a resolution parameter. By tuning the parameter, they were able to obtain the hierarchical communities. [21] defines a tightness function of local community. Similarly, by adjusting a resolution parameter of this function, they obtained the communities at different scales. The hierarchical community detection methods mentioned above all adopt the heuristic optimization of a quality function. Generally, the fact that many real networks have communities with pervasive groups leads to that a global hierarchy of vertices cannot capture the relationships between overlapping vertices. In addition, these hierarchical community detection methods can not reconcile the antagonistic organizing principles of overlapping communities and hierarchy [22]. The combination of soft community memberships and hierarchy may be a visible solution.

To deal with the above problems, we present a novel model-based approach for overlapping and hierarchical community detection. This approach is free to set the number of communities (specified by users or determined automatically), which is beneficial to real-world application scenarios. The generative model contains two parts: the loss function and the *l*_2,1_ norm regularization term, which are balanced by a resolution parameter. The loss function measures the distance between the real network and the expected generated network correlating with the membership of vertices, and the regularization term controls the group sparsity of the model. Thereafter, we derive an update rule of the model parameters to infer the membership matrix of vertices. The resolution parameter balances the loss function and the regularization term. By tuning the balance parameter, we can obtain communities at different scales. When we know the number of communities *K*, it is easy to directly specify *K*. Otherwise, it is easy to specify a large initial *K*_0_, and then the *l*_2,1_ norm regularization term penalizes some empty communities where no vertices participate in. In this way, we get a more accurate number of communities from the large *K*_0_ by abandoning the empty communities.

The rest of the paper is organized as follows. First, we formalize the problems as a generative model and give the update rule of the model parameters. We then verify our approach on various networks including artificial and real-world networks. At last, we summarize the discussions and conclude with our future work.

## Methods

In this section, we first describe our generative model, and then present an algorithm based on nonnegative matrix factorization to learn the parameters of the model. Finally, we offer an illustrative example to depict the main idea of our method.

### Generative model

We consider an undirected and weighted network *G* = (*V*, *E*), where *V* denotes the set of vertices and *E* denotes the set of links. Usually, we use the adjacency matrix **A** to represent *G*, where *A*_*ij*_ equals to the weight of a link between vertex *i* and *j* if they are connected, and otherwise, it is 0. Thus, **A** is a *N* × *N* matrix, where *N* is the number of vertices. In our model, if we know the number of communities *K* in advance, we can set *K* directly. Otherwise, we can set a relative large initial *K*_0_, and then our model will automatically determine a suitable number of communities.

The first step in the model is to generate an expected adjacency matrix $\hat{\text{A}}$, which has the same size as **A**. The element ${\hat{A}}_{ij}$ in $\hat{\text{A}}$ denotes the expected weight of links between vertices *i* and *j*. Here ${\hat{A}}_{ij}$ is specified by a set of parameters *U*_*ik*_ which represents the propensity of vertex *i* belonging to community *k*. Therefore, we define the membership matrix, **U** ∈ ℝ^*N*×*K*^, which consists of all the elements *U*_*ik*_. To be specific, *U*_*ik*_ *U*_*jk*_ denotes the expected weight of links between *i* and *j* in community *k*. Summing over the communities, the expected weight of links between vertices *i* and *j* in the network is:

$$\begin{array}{c} {\hat{A}}_{ij}=\sum_{k=1}^{K}U_{ik}U_{jk}, \end{array}$$ (1)

Eq (1) can also be rewritten as

$$\begin{array}{c} \hat{A}=UU^{T} \end{array}.$$ (2)

Under this model, if vertices *i* and *j* belong to the same community *k*, which means *U*_*ik*_ and *U*_*jk*_ are both relatively large, the value of ${\hat{A}}_{ij}$ will be large. This implies that vertices *i* and *j* have a high propensity of being connected in community *k*. Then, a set of such vertices tends to be connected relatively densely in community *k*. Otherwise, ${\hat{A}}_{ij}$ will be small.

Furthermore, when the number of communities *K* is unknown, it is preferable to determine it automatically. But in practice, it is easy to set an initial maximum number *K*_0_. Then, what we need is to determine *K* from *K*_0_. In the community structure of a network, assuming that a community *k* is “redundant” or unnecessary, it means all the vertices do not participate in this community. Accordingly, the entry *U*_*ik*_ should be zero for all *i*, which implies all the values in the *k*th column of membership matrix **U** will be zero. Finally, the problem becomes how to make some “redundant” columns in the original matrix **U** zero, and select several non-zero columns from **U**. Generally speaking, one usually uses *l*_1_ norm regularization to promote a sparse solution and improve generalization, but it cannot obtain a wide class of solutions known to have certain “group sparsity” structure. Incorporating group information using mixed-norm regularization has been previously discussed in statistics and machine learning. A favorable approach in literature is to use the mixed *l*_2,1_ norm regularization [23, 24]. Inspiring by the above idea, we add a constraint to **U** by using *l*_2,1_ norm, which is defined as

$$\begin{array}{c} {\left\|U\right\|}_{2,1}=\sum_{k=1}^{K}\sqrt{\sum_{i=1}^{N}U_{ik}^{2}}=\sum_{k=1}^{K}\left\|U_{k}\right\|, \end{array}$$ (3)

where **U**_*k*_ is the *k*th column of **U**. As we can see in (3), the *l*_2,1_ norm of a matrix is the sum of vector *l*_2_ norm of its columns, which can be considered as *l*_1_ norm of vector *l*_2_ norm of its columns. So penalization with *l*_2,1_ norm promotes as many zero columns as possible to appear in the matrix. Thus, the *l*_2,1_ norm will achieve the group sparsity.

There are many options to fit the adjacency matrix **A** and the expected adjacency matrix $\hat{\text{A}}$. Least square loss and the generalized Kullback-Leibler (KL) divergence are most widely used [25]. Similar with other NMF-based methods [11–14], we adopt the least square loss between **A** and $\hat{\text{A}}$ for simplicity. Here, we show the motivation of the least square loss from the viewpoint of likelihood. Usually, the observed weight *A*_*ij*_ between vertices *i* and *j* can be represented by the expected weight ${\hat{A}}_{ij}$ adding additional noise **ε**_*ij*_,

$$\begin{array}{c} A_{ij}={\hat{A}}_{ij}+\varepsilon_{ij} \end{array}.$$ (4)

Suppose the noise *ε*_*ij*_ follows zero mean normal distribution with standard deviation of *τ*, that is $A_{ij}∼N({\hat{A}}_{ij},\tau^{2})$. In general, we assume each weight *A*_*ij*_ is independent, therefore, the probability distribution of *A*_*ij*_ conditioned on ${\hat{A}}_{ij}$ is

$$\begin{array}{c} p\left(A_{ij}|{\hat{A}}_{ij}\right)∼\exp\left\{-\frac{{\left(A_{ij}-{\hat{A}}_{ij}\right)}^{2}}{2\tau^{2}}\right\} \end{array}.$$ (5)

Thus for all the weights, the log likelihood can be written as

$$\begin{array}{c} \log\prod_{i=1}^{N}\prod_{j=1}^{N}p\left(A_{ij}|{\hat{A}}_{ij}\right)=-\frac{1}{2\tau^{2}}\sum_{i=1}^{N}\sum_{j=1}^{N}{\left(A_{ij}-{\hat{A}}_{ij}\right)}^{2} \end{array}.$$ (6)

Then maximizing the log likelihood is equivalent to minimize $\overset{N}{\underset{i=1}{\sum }}\overset{N}{\underset{j=1}{\sum }}{\left(A_{ij}-{\hat{A}}_{ij}\right)}^{2}$ in (6). Thus we have

$$\begin{array}{c} \min_{{\hat{A}}_{ij}}\sum_{i=1}^{N}\sum_{j=1}^{N}{\left(A_{ij}-{\hat{A}}_{ij}\right)}^{2}=\min_{U}{\left\|A-{\mathrm{UU}}^{T}\right\|}_{F}^{2} \end{array}.$$ (7)

This means the least square loss underlies the Gaussian additive observation noise [18]. Finally, by adding the *l*_2,1_ norm regularization term to control the group sparsity of **U**, the formulation of our model is

$$\begin{array}{c} \min_{U\geq 0}L\left(A,\hat{A}\right)=\|A-UU^{T}{\|}_{F}^{2}+\lambda {\left\|U\right\|}_{2,1}, \end{array}$$ (8)

where *λ* is a positive real-valued parameter, which controls the degree of group sparsity. In other words, the coefficient *λ* controls the number and the average size of communities. If *λ* is small, we can get smaller communities. And if it is large, we can get less but larger communities. If it is large enough, the whole network becomes a community. Hence, the parameter *λ* tunes the resolution of the network. Different *λ* means various scales of the network.

### Model learning

Firstly we deduce the gradient of (8) with respect to *U*_*ik*_

$$\begin{array}{c} \frac{\partial L}{\partial U_{ik}}={\left(-4\mathrm{AU}+4{\mathrm{UU}}^{T}U\right)}_{ik}+\lambda \frac{U_{ik}}{\sqrt{\sum_{i}U_{ik}^{2}}} \end{array}.$$ (9)

We then get the positive term of (9)

$$\begin{array}{c} {[\cdot ]}_{+}={\left(4{\mathrm{UU}}^{T}U\right)}_{ik}+\frac{\lambda U_{ik}}{\sqrt{\sum_{i}U_{ik}^{2}}}, \end{array}$$ (10)

and the negative term of (9)

$$\begin{array}{c} {[\cdot ]}_{-}={\left(4\mathrm{AU}\right)}_{ik} \end{array}.$$ (11)

According to the gradient decent algorithm, one can use the [⋅]_+_ and [⋅]_−_ to define an iterative learning based update rule as follows:

$$\begin{array}{c} U_{ik}=U_{ik}-\eta_{ik}\frac{\partial L}{\partial U_{ik}}=U_{ik}-\eta_{ik}\left({[\cdot ]}_{+}-{[\cdot ]}_{-}\right) \end{array}.$$ (12)

Here, *η*_*ik*_ is a positive learning rate. If we choose $\eta_{ik}=\frac{U_{ik}}{{\left[\cdot \right]}_{+}}$ according to the Oja rules [26], the update rule becomes a multiplicative update rule:

$$\begin{array}{c} U_{ik}=U_{ik}-\frac{U_{ik}}{{[\cdot ]}_{+}}\left({[\cdot ]}_{+}-{[\cdot ]}_{-}\right)=U_{ik}\frac{{[\cdot ]}_{-}}{{[\cdot ]}_{+}} \end{array}.$$ (13)

Finally, we can simply update *U*_*ik*_ by multiplying its current value with the ratio of the negative term to positive term

$$\begin{array}{c} U_{ik}=U_{ik}\frac{{\left(4\mathrm{AU}\right)}_{ik}}{{\left(4{\mathrm{UU}}^{T}U\right)}_{ik}+\lambda \frac{U_{ik}}{\sqrt{\sum_{i}U_{ik}^{2}}}} \end{array}.$$ (14)

It is worth noting that the update rule converges to a local optimum. But in order to get a better result, we take the similar strategy as other NMF methods used. We first initialize ten “seeds” randomly, that is, we initialize ten **U** matrices randomly. When the algorithm converges, the ten different matrices will lead to ten different results, which corresponds to ten values of the loss function respectively. We can then find the minimum value from these values, and consider the corresponding matrix **U** as the final solution that will be not too far from the global optimum.

Both of disjoint and overlapping communities can be derived from the obtained membership matrix **U**. To be specific, to derive a disjoint partition, vertex *i* can be assigned to community *r* = *argmax*_*k*_{*U*_*ik*_, *k* = 1, 2, …, *K*}. To construct a structure with overlapping communities, we take the similar strategy in [12] and [27]. We first choose the maximum and minimum value in vector {*U*_*i*1_, *U*_*i*2_, …, *U*_*iK*_},

$$\begin{array}{c} U_{ip}=\max_{U_{ik}}\left\{U_{ik},k=1,2,\ldots ,K\right\}, \end{array}$$ (15)

$$\begin{array}{c} U_{iq}=\min_{U_{ik}}\left\{U_{ik},k=1,2,\ldots ,K\right\} \end{array}.$$ (16)

We then rescale each entry in this vector to [0–1] as follows,

$$\begin{array}{c} U_{ik}^{new}=\frac{U_{ik}-U_{iq}}{U_{ip}-U_{iq}},k=1,2,\ldots ,K \end{array}.$$ (17)

In this way, the maximum value in each row rescales to 1 and the minimum value in each row rescales to 0. The rest entries rescale to values in the range of 0 to 1. We then vary a threshold from 0 to 1 and set all those entries in **U** that exceed a predetermined threshold to 1, and 0 otherwise. Now different thresholds will lead to different communities. In order to get the desired communities, we select modularity *Q* (or minimum description length *L*) as the quality metric, which depends on the specialized scenarios. Thus, the proper threshold is the one that corresponds to the community structure with the maximum *Q*-value (or the minimum *L*-value).

Now, inspired by the proof in [11], we will give the proof of the correctness of the updating rules in (14).

THEOREM 1. At convergence, the converged solution **U** of the updating rule in (14) satisfies the Karush-Kuhn-Tucker (KKT) condition of the optimization theory [28].

PROOF. Because of the nonnegativity constraint of **U**, we introduce the Lagrangian multiplier **δ** for **U**. In this way, we can construct the Lagrangian function as

$$\begin{array}{c} L\prime =\|A-{\mathrm{UU}}^{T}{\|}_{F}^{2}+\lambda {\left\|U\right\|}_{2,1}-tr\left(\delta U^{T}\right), \end{array}$$ (18)

where **δ** is a *N* × *K* nonnegative matrix and its element *δ*_*ik*_ is the Lagrangian multiplier of *U*_*ik*_. Then we have the KKT conditions

$$\begin{array}{c} \left\{ \begin{array}{c} \frac{\partial L\prime }{\partial U_{ik}}={\left(-4\mathrm{AU}+4{\mathrm{UU}}^{T}U\right)}_{ik}+\lambda \frac{U_{ik}}{\sqrt{\sum_{i}U_{ik}^{2}}}-\delta_{ik}=0\\ U_{ik}\geq 0\\ \delta_{ik}\times U_{ik}=0\\ \delta_{ik}\geq 0\\ \forall i,k \end{array}\right. \end{array}.$$ (19)

Following the first KKT condition, we obtain

$$\begin{array}{c} \frac{\partial L\prime }{\partial U_{ik}}U_{ik}=\left\{{\left(-4\mathrm{AU}+4{\mathrm{UU}}^{T}U\right)}_{ik}+\lambda \frac{U_{ik}}{\sqrt{\sum_{i}U_{ik}^{2}}}\right\}U_{ik}-\delta_{ik}U_{ik}=0 \end{array}.$$ (20)

Further, by the third KKT condition, we have

$$\begin{array}{c} \left\{{\left(-4\mathrm{AU}+4{\mathrm{UU}}^{T}U\right)}_{ik}+\lambda \frac{U_{ik}}{\sqrt{\sum_{i}U_{ik}^{2}}}\right\}U_{ik}=0 \end{array}.$$ (21)

On the other hand, once **U** converges, according to the updating rule of (14), the converged solution **U** satisfies

$$\begin{array}{c} U_{ik}=U_{ik}\frac{{\left(4\mathrm{AU}\right)}_{ik}}{{\left(4{\mathrm{UU}}^{T}U\right)}_{ik}+\lambda \frac{U_{ik}}{\sqrt{\sum_{i}U_{ik}^{2}}}}, \end{array}$$ (22)

which can be written as

$$\begin{array}{c} \left\{{\left(-4\mathrm{AU}+4{\mathrm{UU}}^{T}U\right)}_{ik}+\lambda \frac{U_{ik}}{\sqrt{\sum_{i}U_{ik}^{2}}}\right\}U_{ik}=0 \end{array}.$$ (23)

This is identical to (21). Besides, the updating rule guarantees the nonnegativity of **U** and **δ** is the Lagrangian multipliers, and thus the second and the fourth KKT conditions are satisfied. Then the updating rule in (14) satisfies all the above KKT conditions, so we finish the proof of the correctness of our updating rule.

### An illustrative example for *l*_2,1_ norm regularization

**Fig 1:**
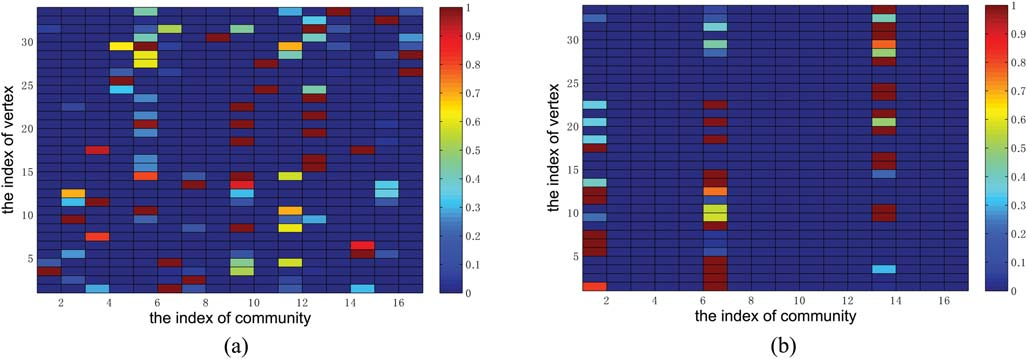
An illustrative example for depicting the main effect of the *l*_2,1_ norm regularization term in our method on karate club network. (a) the color mapping of the membership matrix **U** obtained by standard NMF. (b) the color mapping of the membership matrix **U** obtained by our method. X-axis represents the index of community, and Y-axis represents the index of vertex. Colors close to red indicate strong propensity of vertex *i* belonging to community *k*.

In this section, we do not intend to show the whole procedure of our method, but to depict the main effect of our above method by adding *l*_2,1_ norm regularization. Here we use the well-known karate club network, and assume the number of communities is 17 at first. Fig 1(a) is the color mapping of **U** normalized to 0–1 obtained by standard NMF [25], where colors close to red indicate the strong propensity of vertex *i* belonging to community *k*, and colors close to blue indicate the weak propensity of vertex *i* belonging to community *k*. Fig 1(b) is the color mapping of **U** normalized to 0–1 obtained by our method. Here, we set the resolution parameter *λ* = 1.7, and in the later sections we will introduce how to determine *λ* in details. As we can see, both the standard NMF and our method will get the membership matrix **U** with size of 34 × 8. However, for our method, there are only three non-zero columns, and the remaining columns are all zeros. This suggests that, although we set the number of communities at a large number at the beginning, but actually, not all communities are essential for this network. It also means that there are some communities in which all the vertices do not involved, and the three non-zero columns are the derived three communities that we are looking for. As a result, after removing the unnecessary communities, we get the number of communities as *K* = 3, corresponding to 3 significant communities. But for the standard NMF without group sparsity, when we set *K* = 17, it cannot select the suitable communities from the initial setting. The vertices are assigned to the 17 communities, and hence we cannot get the significant split of the Karate club network.

It is worth noting that we can get both the overlapping and disjoint communities from **U**. The strategy about how to use the membership matrix **U** to determine the communities and how to choose a suitable threshold have been introduced in the Model Learning section, which are similar with other NMF methods. Besides, we can also get the multi-scale structures when varying the resolution parameter *λ*.

## Results

In this section, we test the performance of our approach on artificial and real-world networks in terms of the results of hard-partitions, overlapping structures, as well as hierarchical structures. We also give the analysis of the resolution parameter in this model.

The methods compared include: Louvain method [20] and Infomap [29], both of which are the most popular hard-partitioning methods; CPM [4], which is one of the most widely used overlapping community detection method; Fuzzy Infomap (F-Infomap) [30], which is an extension of Infomap to detect overlapping communities; SNMF [11] and BNMTF [12], which are the model-based methods based on NMF. Except some special comparison scenarios, we will make a full comparison against all these above methods.

There are various quality metrics that are used to evaluate the goodness of community structures. However, each of these metrics is only applicable to several types of evaluation scenarios. For this reason, we will use different quality metrics in different cases. They include: normalized mutual information (NMI) [31] which is used to evaluate the hard-partition results on networks with known community structures, generalized normalized mutual information (GNMI) [19] which is used to evaluate the overlapping results on networks with known community structures, modularity *Q* [32] which can be used to evaluate the hard-partition results on real networks without ground-truth, and the extended map equation *L* [30] which can be used to evaluate overlapping results on real networks without ground-truth.

### Test on synthetic networks

To evaluate the performance of our approach, we conduct experiments on three types of synthetic benchmarks.

We firstly evaluate the hard-partitioning results. Here we adopt the widely used Newman’s model, proposed in [3]. The graph consists of 128 vertices, which are divided into four communities of 32 vertices each. Each vertex has the expected degree 16, including an average *z*_*in*_ edges connecting to vertices within the same community and *z*_*out*_ edges to vertices in other communities. With the increase of *z*_*out*_, the community structure becomes more and more ambiguous. In this experiment, we first choose Louvain method, Infomap, SNMF and BNMTF to be compared, and use NMI as the accuracy metric. The higher the value of NMI index, the better the results will be. Because CPM and F-Infomap only provide overlapping community results, we cannot compute their NMI values, and hence we cannot compare with them in terms of NMI. For this problem, we further use GNMI which is suitable for evaluating both overlapping and disjoint structures as the accuracy metric to compare with all these methods.

**Fig 2:**
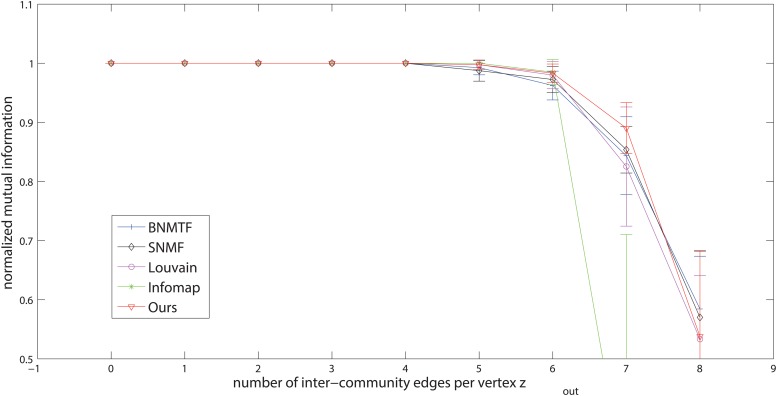
Evaluation of different methods on Newman’s benchmark networks in terms of NMI.

**Fig 3:**
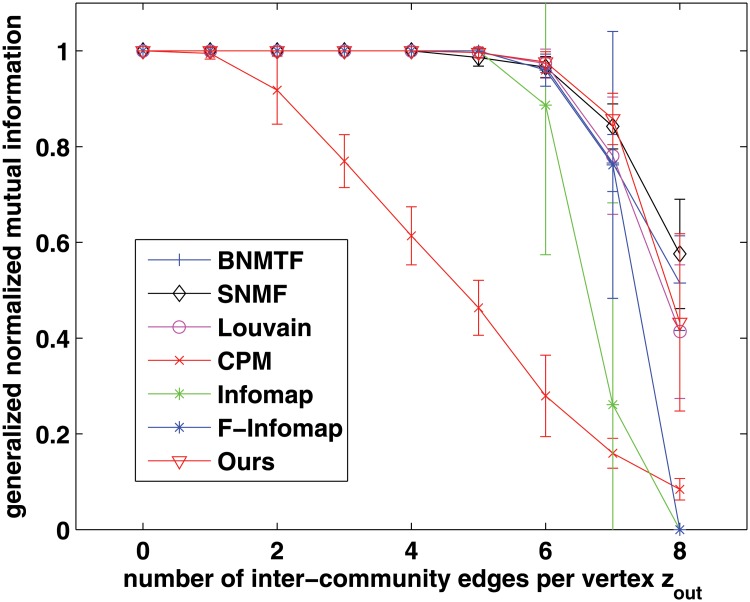
Evaluation of different methods on Newman’s benchmark networks in terms of GNMI.

The comparisons in terms of NMI and GNMI are shown in Figs 2 and 3, respectively. As we can see, when *z*_*out*_ is small, the community structures are very clear, so both of the NMI and GNMI accuracies of all the methods are very high, close to 1. With the increase of *z*_*out*_, the community structures will be not so clear, and it becomes a challenge to these methods. Especially, when *z*_*out*_ > 6, the NMI (and GNMI) accuracy begins to decrease quickly. When *z*_*out*_ > 8, which means the number of between-community edges per vertex is more than that of within-community edges and there is almost no community structure in the network, it will lead to a very low value of NMI (and GNMI) index for most of the methods. But in general, the performance of our method is often better than (or competitive with) that of the other methods in terms of both NMI and GNMI.

We then evaluate the overlapping community results. Here we adopt a new type of benchmark proposed by Lancichinetti, Fortunato and Radicchi [33], named as LFR. Compared with Newman’s model, LFR model can not only generate overlapping communities, but also possess the statistical property of heterogeneous distributions of degree and community size, which often appears in real-world networks.

Following the experiment designed by Lancichinettic *et al.* [33], the setting of the parameters in LFR model is shown in Table 1. Here, *N* denotes the number of vertices; *k* denotes the average degree per vertex; *maxk* denotes the maximum degree of vertex; *u* denotes the mixing parameter, i.e., each vertex shares a fraction *u* of its links with vertices in other communities; *t*_1_ denotes the minus exponent for the degree sequence; *t*_2_ denotes the minus exponent for the community size distribution; *minc* denotes the minimum for the community size; *maxc* denotes the maximum for the community size; *on* denotes the fraction of overlapping vertices; *om* denotes the number of memberships of the overlapping vertices. In Table 1, we use S denotes the benchmark networks with smaller communities, B denotes the the benchmark networks with larger communities; 0.1 and 0.3 denotes networks with different mixing parameters which are 0.1 and 0.3, respectively. So we produced four types of LFR benchmarks.

**Table 1:** The detailed parameters of LFR benchmark networks.

Network	*N*	*k*	*maxk*	*u*	*t*_1_	*t*_2_	*minc*	*maxc*	*on*	*om*
0.1S	1000	20	50	0.1	2	1	10	50	0–0.5	2
0.3S	1000	20	50	0.3	2	1	10	50	0–0.5	2
0.1B	1000	20	50	0.1	2	1	20	100	0–0.5	2
0.3B	1000	20	50	0.3	2	1	20	100	0–0.5	2

**Fig 4:**
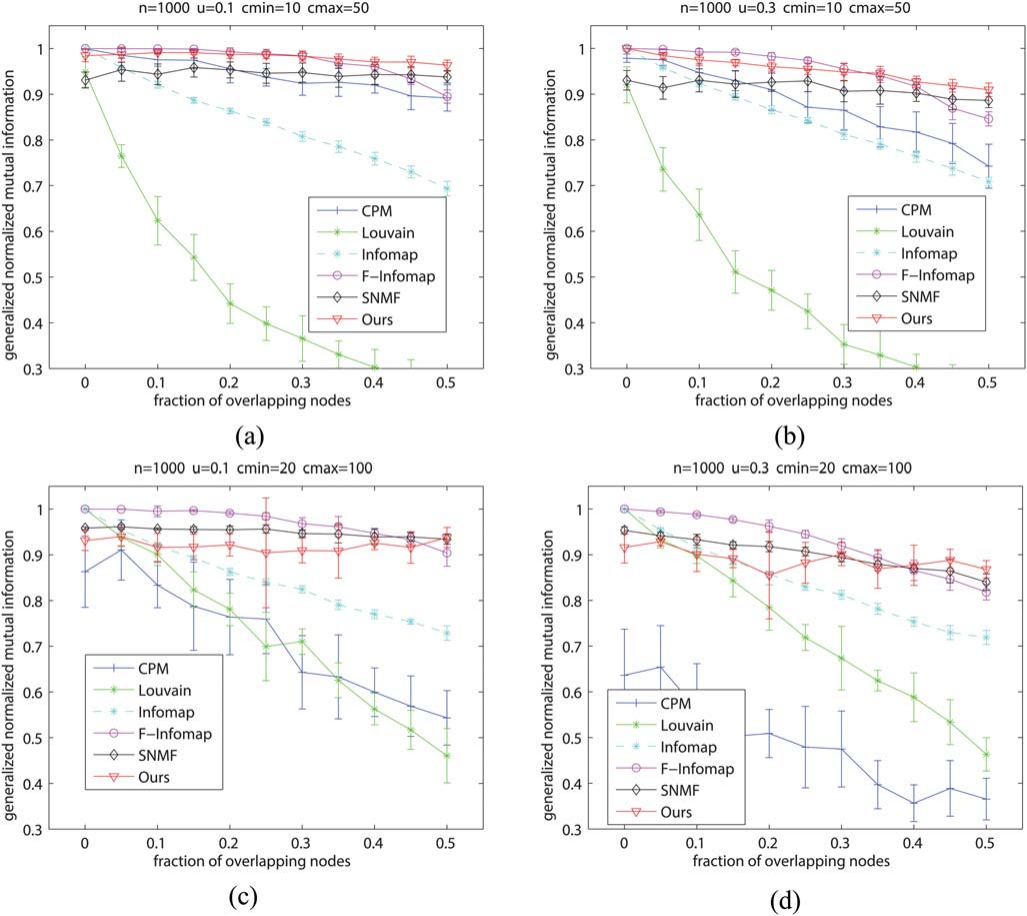
Evaluation of different methods on LFR benchmark networks. (a) Comparison on LFR networks with small mixing parameter and small communities; (b) Comparison on LFR networks with large mixing parameter and small communities; (c) Comparison on LFR networks with small mixing parameter and large communities; (d) Comparison on LFR networks with large mixing parameter and large communities.

In this experiment, we choose Louvain method, CPM, Infomap, F-Infomap and SNMF to be compared, and use GNMI as the accuracy metric. Because BNMTF cannot provide results within 100h for each set of the tests, we did not include it here. The comparison results are shown in Fig 4. As we can see, F-Infomap and our method perform best on networks with small communities (see Fig 4(a) and 4(b)). On large communities (see Fig 4(c) and 4(d)), the performance of our method is competitive with that of F-Infomap and SNMF, but much better than that of the other 3 methods. With the increase of the fraction of overlapping vertices *on*, the overlapping community structure will become more and more indistinct, and hence the curves will often decrease. But compared with other methods, our approach is still relatively stable with the change of *on*, and performs well. Besides, the performance of our approach is also relatively stable when the mixing parameter *u* changes from 0.1 to 0.3. This also expresses the effectiveness of our method on LFR benchmarks.

Finally we evaluate the hierarchical community results. We adopt the hierarchical community model proposed by Lancichinetti *et al.* [19], which extends Newman’s basic model to accommodate the hierarchical structures. This new model contains 512 vertices and two levels in all. At its second level, the 512 vertices are arranged in 16 communities of 32 vertices each. Further, the 16 communities form 4 super communities of 128 vertices each at its first level. On average, each vertex has *k*_1_ links connecting other 31 vertices within the same community at the second level, has *k*_2_ links connecting the other 96 vertices within the same supercommunity at the first level, and has *k*_3_ links connecting the rest of the network. Usually, with the increase of *k*_2_ and *k*_3_, the communities at each level become more and more unclear, leading to a challenge for all the methods. Following the parameter configuration in [19], here we specify *k*_1_ = *k*_2_ = 16 and vary *k*_3_ from 16 to 36 with an interval of 2.

**Fig 5:**
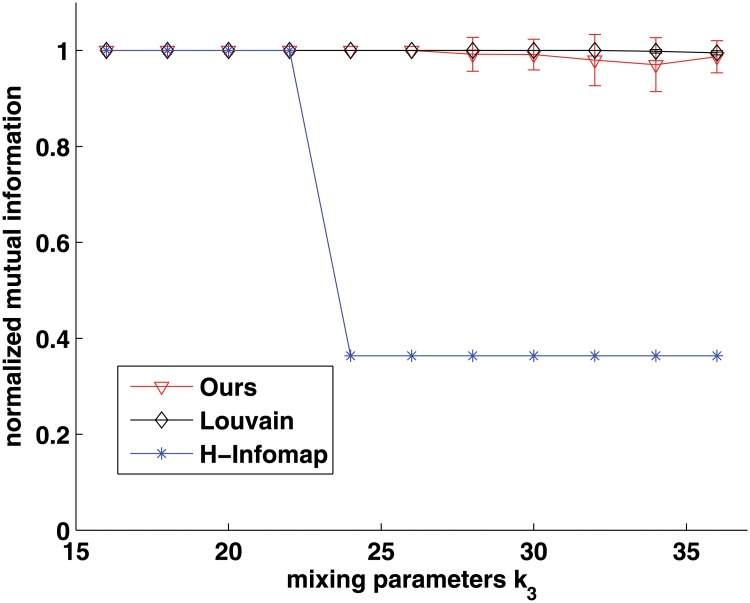
Comparison with Louvain method and H-Infomap on hierarchical community benchmarks at its first level. X-axis indicates the mixing parameter, and the Y-axis indicates the normalized mutual information.

**Fig 6:**
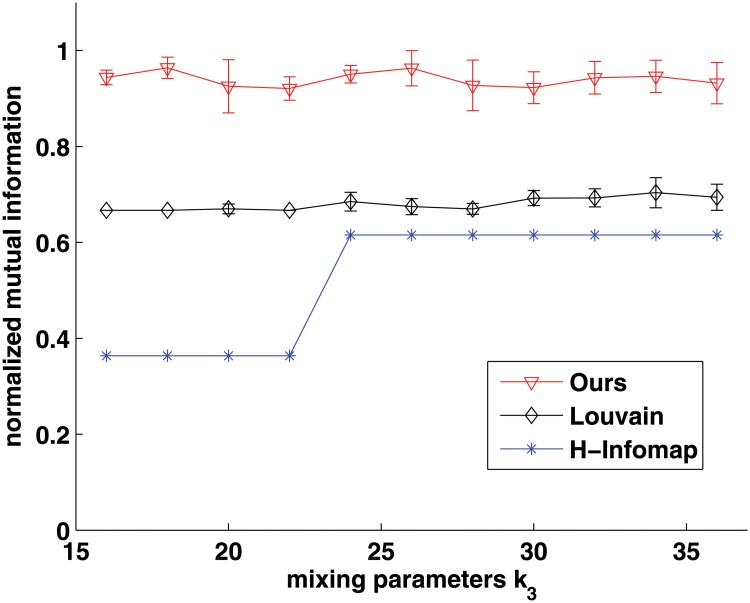
Comparison with Louvain method and H-Infomap on hierarchical community benchmarks at its second level. X-axis indicates the mixing parameter, and the Y-axis indicates the normalized mutual information.

As this model only provides the ground-truth of hierarchical structure with disjoint communities, hence we choose Louvain method which can provide hierarchical community structure to be compared. Besides, because Informap has been extended to detect hierarchical structure [34], we also choose it to be compared, and use the name H-Infomap in short. Fig 5 shows this comparison results at the first scale. As we can see, the performance of our approach is better than that of H-Infomap, and is competitive with that of Louvain method. Especially, when *k*_3_ > 26, the NMI accuracy of Louvain method is slightly better than that of our method, although they are both very high and close to 1. Then when *k*_3_ > 22, because H-Infomap detects 512 communities, which is much more than the ground-truth, its value of NMI decreases to around 0.35. Furthermore, Fig 6 shows the comparison results at the second level. As we can see, the performance of our approach is better than that of both Louvain method and H-Infomap. The reason may be that, Louvain method is based on the modularity optimization suffering from resolution limits [35], and thus it cannot find a high resolution solution, such as the structure at the second level structure with 16 communities. H-Infomap also does not find the real number of communities 16 at the second level, e.g., H-Infomap often finds 4 or 512 communities on the networks. On the contrary, our method is flexible to adjust the resolution parameter *λ*, and hence can accurately detect community structures at different levels.

To sum up, we adopt three types of artificial benchmark networks to test the performance of our approach. The results not only show the superior performance of our approach, but also validate its flexible ability to detect communities in terms of different cases, such as disjoint communities, overlapping communities as well as the hierarchical structures.

### Test on real-world networks

As real networks may have some different topological properties from artificial networks, here we use various real-world networks to further evaluate the performance of our algorithm. We select 8 widely-used real networks which are summarized in Table 2, where *N* denotes the number of vertices, *M* denotes the number of links, and *K* denotes the number of communities. Especially, “—” implies that the number of communities is unknown. In the following, we will test the performance of different algorithms in terms of disjoint communities and overlapping communities, respectively.

**Table 2:** Real-world networks used here.

Datasets	*N*	*M*	*K*	Descriptions
Karate	34	78	2	Zachary’s karate club [36]
Dolphins	62	159	2	Dolphin social network [37]
Polbooks	105	441	3	Books about US politics [38]
Word	112	425	2	Word network [39]
Football	115	613	12	American college football [3]
Les Miserables	77	254	-	the novel by Victor Hugo [40]
C. elegans neural	297	2148	-	the neural network of C. elegans [1]
C. elegans metabolic	453	2025	-	the metabolic network of C. elegans [41]

Firstly, we estimate the disjoint community results for different algorithms. Because Table 2 contains networks with and without known communities, here we take modularity *Q* as a unified quality metric. In this experiment, we select Louvain method, Infomap, BNMF and BNMTF to be compared. CPM and F-Infomap cannot provide the hard-partitioning results, and hence we cannot compute *Q*-values for these results. Therefore, we did not include these two methods here. The comparison results are shown in Table 3. The last row of Table 3 is the average *Q*-value of each method on all the networks. Because Louvain method is based on the optimization of modularity *Q*, it is not surprising that it gives the best performance on almost all the networks. Thus, for clarity, we show the results of Louvain method in the right column of the table separately. Except Louvain method, we mark the best results by **boldface** and our second best results by *italic*. As we can see, our approach achieves the best performance among those methods which do not directly optimize modularity *Q*. Also of note is that, our approach has the advantage of providing overlapping and hierarchical solutions, while other methods do not.

**Table 3:** Comparison with other methods in terms of modularity (Note that we mark the best results by boldface and our second best results by *italic*).

Modularity *Q*	Infomap	SNMF	BNMTF	Ours	Louvain
Karate	0.4020	0.3715	0.3715	**0.4081**	0.4188
Dolphins	0.5108	0.3899	0.3848	**0.5054**	0.5286
Polbooks	**0.5268**	0.5093	0.0548	*0.5126*	0.4986
Word	0.0138	0.1577	0.1978	**0.2712**	0.2906
Football	0.6007	0.5796	0.6005	**0.6011**	0.6046
Les Miserables	0.5391	**0.5466**	0.5460	0.5437	0.5556
C. elegans neural	**0.3920**	0.3400	0.3306	*0.3378*	0.3876
C. elegans metabolic	**0.3880**	0.3060	0.3252	*0.3631*	0.4266
Average *Q*	0.4216	0.4001	0.3514	**0.4429**	0.4639

Next, we estimate the qualities of overlapping communities obtained by different algorithms, and select the extended map equation *L* as the quality metric. Here we use all the methods to be compared. The results are shown in Table 4. The last row of Table 4 is the average *L*-value of each method on all the networks. Because both F-Infomap and Infomap directly optimize the Minimum Description Length *L*, it is not surprising that they achieve the best and second best performance, respectively. Therefore, for clarity, we put their results on the right side of the table separately. As we can see, on average our approach achieves the best performance among those methods which are not based on the optimization of function *L*. To sum up, these two experiments, in terms of disjoint and overlapping communities, both validate the effectiveness of our method.

**Table 4:** Comparison with other methods in terms of the map equation *L* for overlapping communities (Note that we mark the best results by boldface and our second best results by *italic*).

Extended map equation *L*	Louvain	CPM	SNMF	BNMTF	Ours	Infomap	F-Infomap
Karate	4.3359	5.0634	4.4093	4.4093	**4.2510**	4.3118	4.2574
Dolphins	4.9104	5.6240	5.1258	5.6084	**4.8566**	4.8854	4.8296
Polbooks	5.5837	5.8534	5.5631	5.5696	**5.4701**	5.4669	5.4392
Word	6.5642	**6.5431**	6.7887	6.6628	6.6308	6.3431	6.3297
Football	5.4982	5.5204	5.4467	**5.4429**	*5.4467*	5.4467	5.4429
Les Miserables	**4.7632**	5.2518	4.8907	4.8845	*4.8238*	4.6813	4.6169
C. elegans neural	7.6309	7.9940	7.6768	**7.6180**	*7.6263*	7.5323	7.4900
C. elegans metabolic	7.4736	7.6469	7.6620	7.4129	**7.3692**	7.2498	7.1415
Average *L*	5.8450	6.1871	5.9454	5.9511	**5.8093**	5.7397	5.6934

**Fig 7:**
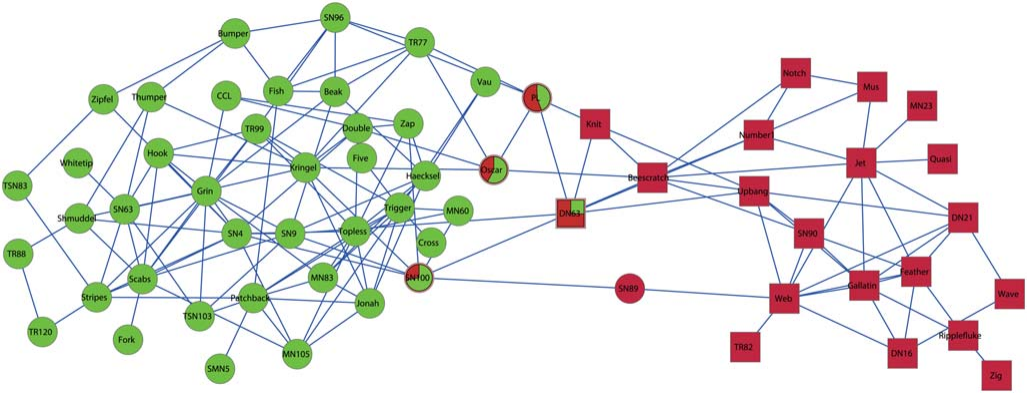
Two communities detected by our approach on dolphins network. Here different shapes represent the ground-truth communities, and different colors represent the communities obtained by our approach. Especially, the overlapping vertices are shown by pie vertices.

**Fig 8:**
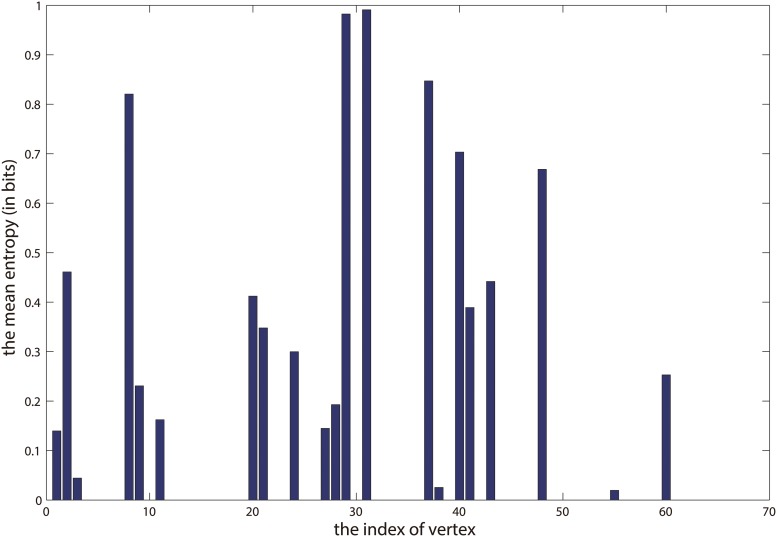
Four communities detected by our approach on dolphins network. Here different shapes represent the ground-truth communities, and different colors represent the communities obtained by our approach. Especially, the overlapping vertices are shown by pie vertices.

In particular, here we take the dolphins social network as an example to test the performance of our approach in more details. The dolphins social network was reported by Lusseau [37], where vertices represented the dolphins and a link was created if two dolphins were observed together more often than expected by chance from 1994 to 2001. In the regular experiment, the network is often divided into two communities, where one community mainly consists of male dolphins and the other mainly consists of female dolphins, which are marked by square and cycle vertices, respectively (see Figs 7 and 8).

**Fig 9:**
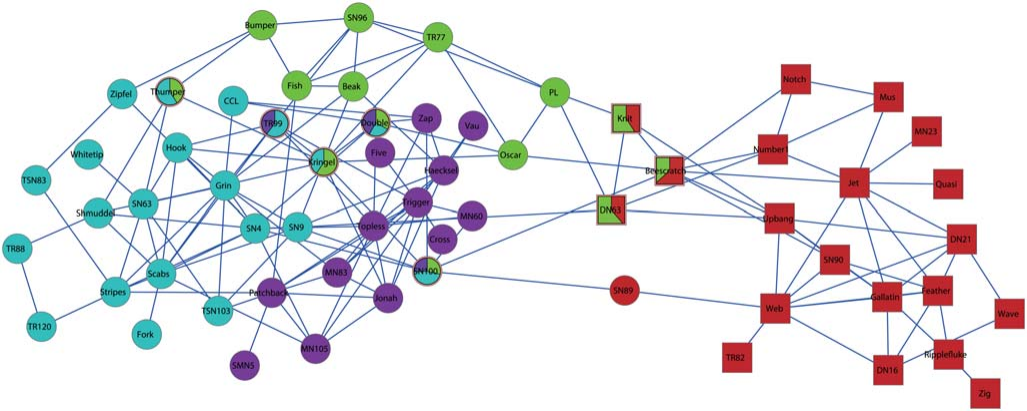
The mean entropy of membership distribution when *K* = 2. X-axis indicates the index of each vertex, and the Y-axis indicates the mean entropy of membership distribution when *K* = 2.

**Fig 10:**
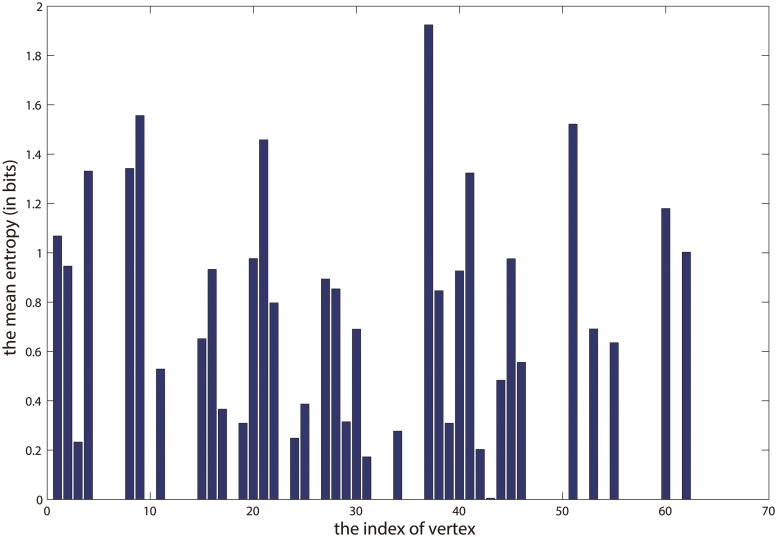
The mean entropy of membership distribution when *K* = 4. X-axis indicates the index of each vertex, and the Y-axis indicates the mean entropy of membership distribution when *K* = 4.

Fig 7 illustrates the two communities detected by our approach at the scale of *λ* = 1.7. Our result is marked by different colors, which successfully discovers the two communities with four overlapping vertices: *PL*, *Oscar*, *DN63*, and *SN100*. Moreover, as mentioned in [10], one can measure the uncertainty in assigning vertices to communities by the mean entropy, so that they used it to monitor the allocation confidence. Besides, [11] takes a similar strategy, and they also used the mean entropy to infer how active (meaning the degree of uncertain or fuzzy) of a vertex is. According to these literatures, since each row of **U** in our model represents the propensity that a vertex belongs to the communities, we can normalize each row of **U** to make its summation to be 1. In this way, the propensity that a vertex belongs to the communities can be considered as a probability distribution. So we can use this to compute the entropy of this vertex, and then measure its uncertainty in terms of the community memberships. High value of entropy stands for high uncertainty, which also means this vertex is more active than other vertices. Therefore, here we monitor the allocation confidence in the viewpoint of the mean entropy (in bits), which is defined as

$$\begin{array}{c} H=-\sum_{k}U_{ik}\log_{2}U_{ik}, \end{array}$$ (24)

and we use it to infer the activities of the dolphins in the communities. Fig 9 shows the mean entropy of membership distribution of the result in Fig 7. It is noteworthy that the four highest spikes in Fig 9 correspond to the four overlapping vertices in Fig 7. This suggests that these overlapping vertices are more active than others, and also they are located next to the junction of the two communities. Hence it makes sense that they are allocated to both of the two communities. Furthermore, Fig 8 illustrates the four communities at the scale of *λ* = 1.5. As we can see, it reveals the community structure at a higher resolution, and that is the larger community in Fig 7 are further divided into 3 smaller communities here. It is known that, the basic partition of dolphins network is based on the gender, and the larger community in Fig 7 mainly consists of female dolphins. However, it is observed that two smaller communities consisting of male dolphins in Fig 8 are nested in the larger community in Fig 7. For example, in the community marked by green in Fig 8, the vertices such as *PL*, *Oscar*, *SN96*, *Beak*, and *Bumper* are all male dolphins. Again, in the community marked by purple in this figure, *MN60*, *Cross*, *Topless*, *Haecksel*, *Jonah*, and *MN105* are also male dolphins. So it demonstrates that, compared with community detection at only one scale, the detection of communities at multi-scales can provide more detailed information to analyze the network. Similar with the result in Fig 7, here the overlapping vertices are also near the boundaries of different communities. Again, we plot the mean entropy of membership distribution of result in Fig 8, which is shown in Fig 10. In this figure, the four highest spikes correspond to the *SN100*, *Double*, *TR99*, and *Kringel*, respectively, which are all the overlapping vertices. Especially we find out that, the mean entropy in Fig 9 is much sparser than that in Fig 10. This may imply that the vertices in smaller communities are usually more active than those in larger communities.

### Analysis of the resolution parameter

Here we analyze the resolution parameter *λ* in the model. This resolution parameter controls the weight of the regularization term in (8). With the increase of *λ*, the importance of the regularization term will increase. Especially, when *λ* equals to 0, the regularization term will have no effect on this model.

**Fig 11:**
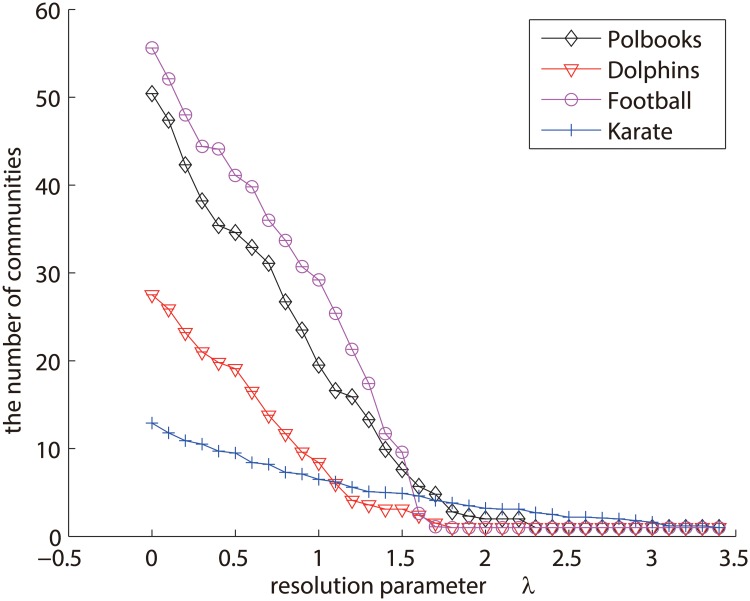
The number of communities at different scales. X-axis indicates the different *λ*, and Y-axis indicates the number of communities.

In particular, the larger the resolution parameter, the sparser the membership matrix **U**. Here we take four real-world networks as an example, which are *polbooks*, *dolphins*, *football*, and *karate* networks. Firstly, we present the relationship between the resolution parameter *λ* and the number of communities in Fig 11. As we can see, the number of communities will decrease with the increase of *λ*. This is because the regularization term will penalize **U**, making it become sparser with the increase of *λ*. As a result, more “redundant” communities will be abandoned, and hence the network will consist of only few communities with large size. Especially, when *λ* > 3, there will be only one community for each of the networks. Similar with other hierarchical methods, sticking to a resolution can get the corresponding community structure at that scale. In general, it is easy to set a large resolution parameter firstly, to get the community structure at low scale first. And then, by decreasing the resolution parameter gradually, one can explore the hierarchical community structures at high scales. Finally, the natural hierarchy of the network will be detected.

**Fig 12:**
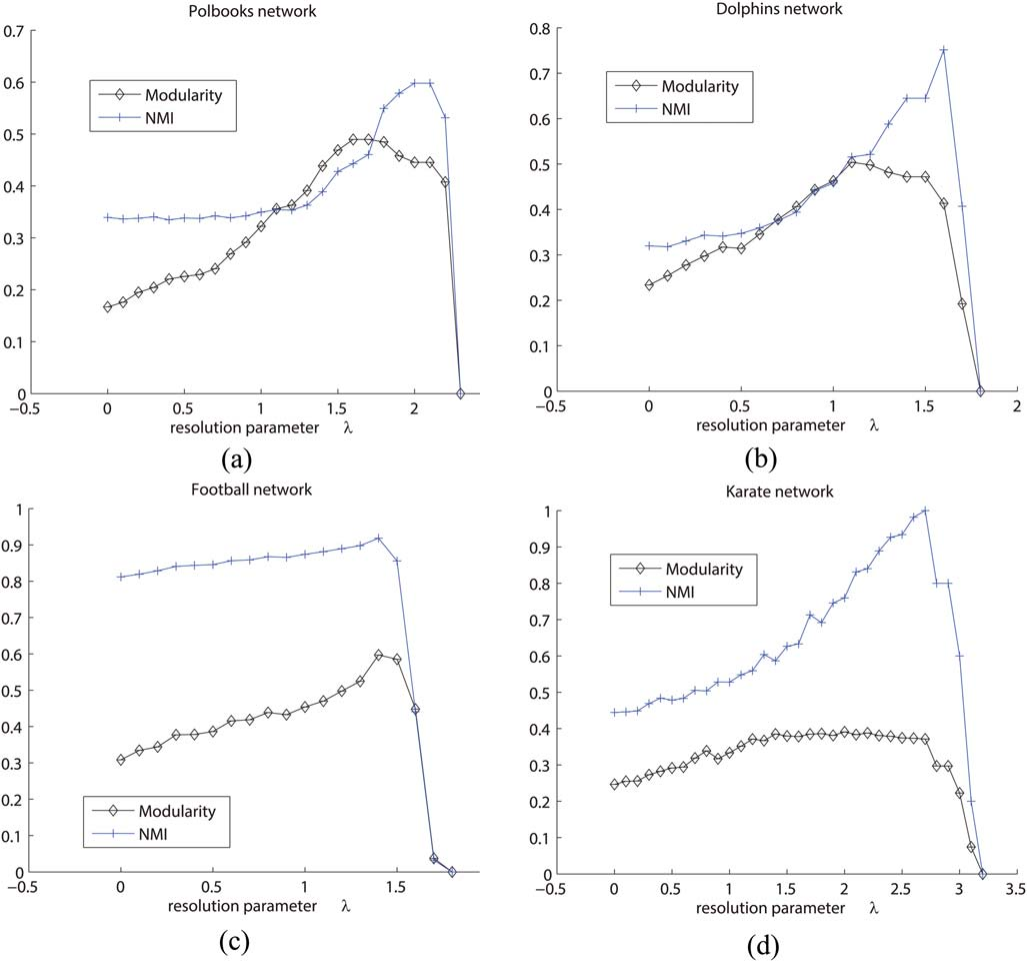
The qualities of communities at different scales. X-axis indicates the resolution parameter *λ*, and Y-axis indicates the modularity and normalized mutual information (NMI) (a) The values of modularity and NMI on polbooks network; (b) The values of modularity and NMI on dolphins network; (c) The values of modularity and NMI on football network; (d) The values of modularity and NMI on karate network.

Furthermore, we check the qualities of community structures at different scales. Fig 12 represents the results of these networks in terms of NMI and modularity *Q*, respectively. In general, the trend of community qualities for each of the networks is like a parabola, which increases from a low quality to a high value, and then falls down again. The phenomenon is intuitive. This is because the small resolution parameter leads to many but very small communities which often do not meet the criteria of well-defined communities. With the increase of *λ*, the quality of its community structure will become better and better. But finally, because large resolution parameter often strongly constrains the sparsity of **U**, the whole network will become one community at last, which leads to low values of modularity and NMI again. Also, the actual number of communities often corresponds to the largest NMI accuracy. For example, the largest NMI accuracy of dolphins network appears when the resolution parameter is around 1.6, where the number of communities is 2, shown in Figs 11 and 12(a). The largest NMI accuracy of karate network appears when the resolution parameter is around 2.6 where the number of communities is 2 in Figs 11 and 12(b). The same phenomenon also happens on the other two networks.

**Fig 13:**
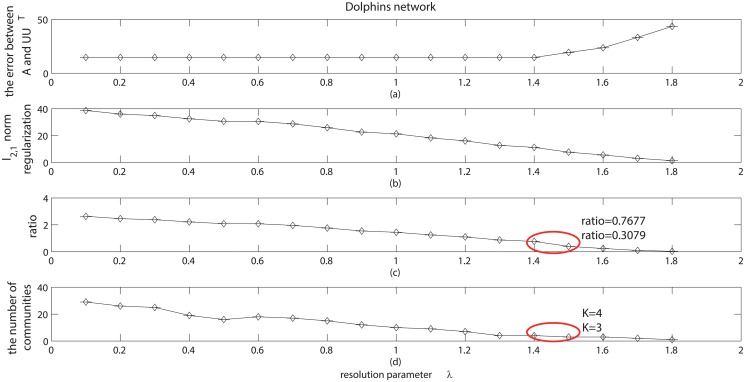
Resolution parameter analysis on dolphins network. (a) shows the error between the adjacency matrix **A** and the expected adjacency matrix **UU** ^*T*^ with different resolution parameter *λ*. (b) shows the value of *l*_2,1_ regularization term with different resolution parameter *λ*. (c) shows the ratio of *l*_2,1_ regularization term to the error between **A** and **UU** ^*T*^. (d) shows the number of communities with different resolution parameter *λ*.

**Fig 14:**
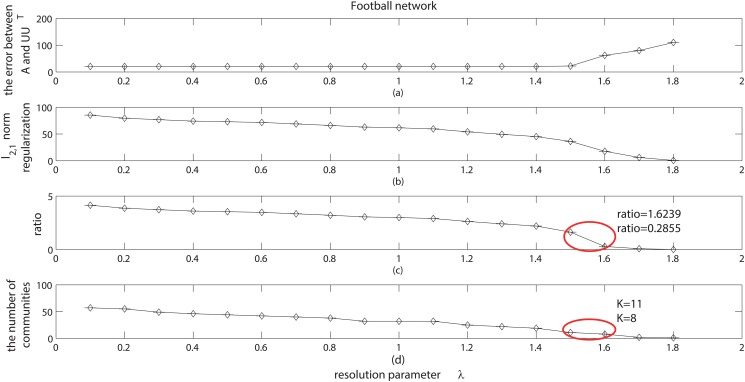
Resolution parameter analysis on football network. (a) shows the error between the adjacency matrix **A** and the expected adjacency matrix **UU** ^*T*^ with different resolution parameter *λ*. (b) shows the value of *l*_2,1_ regularization term with different resolution parameter *λ*. (c) shows the ratio of *l*_2,1_ regularization term to the error between **A** and **UU** ^*T*^. (d) shows the number of communities with different resolution parameter *λ*.

As discussed in [19] and [21], if one wants to get the hierarchical community structure, one often has to scan across the resolution parameter. But here, we tend to find a “robust” region for this parameter, which can give the satisfactory results in most cases. There have been several strategies to determine resolution parameters, such as the cross validation, the consensus clustering, and so on. Of particular interest is the novel viewpoint proposed by [42–44], which focused on a dynamic process to explore the networks. They provided a physical interpretation of the resolution parameter (the inverse of time *t*), and then gave the robustness of partitions at time *t*. But these strategies are not suitable for our model. Here we give a new strategy. According to our experiments, with the increase of the resolution parameter *λ*, the error between the adjacency matrix **A** and the expected adjacency matrix **UU** ^*T*^ (the first term of (8)) will usually increase (see Figs 13(a) and 14(a)), and the *l*_2,1_ norm regularization (the second term of (8)) will usually decrease (see Figs 13(b) and 14(b)). This means that, with the increase of *λ*, the regularization term will make **U** sparser, which will lead to a smaller value of *l*_2,1_norm of **U** and produce more zero columns in **U**. On the other hand, a sparser **U** will also result in a larger reconstructed error, and hence the error between the adjacency matrix **A** and the expected adjacency matrix **UU** ^*T*^ will be larger. In the following, we tend to find a good balance between these two terms. We depict the ratio of *l*_2,1_ norm regularization term to the error term in Fig 13(c) and 13(c), and express the number of communities obtained at different scales in Figs 13(d) and 14(d). As shown, when this ratio is around 0.5 (marked by the red ellipse), which means the value of the *l*_2,1_ norm regularization term in (8) is about half of value of the error regularization term, we can get a suitable number of communities close to the real number of communities. In this situation, we will often get the satisfactory community results. Thus, in general we recommend the “robust” region for the resolution parameter to be a value making the ratio of the second terms to the first terms in (8) around 0.5. This is also the setting in our experiments.

## Application

We use the network of word associations as an application in this section, to demonstrate the superior performance of our approach in solving real-world problems. This network was created by the University of South Florida and University of Kansas [6], which included 5,019 stimulus words. There were in all more than 6,000 participants joining in this project. And they were asked to write the first word in their minds once they heard a word. In this way, the word association network containing 5,017 vertices and 29,148 links was constructed. Originally, the network was weighted where the weight of link represents how frequently two given words were associated. However, we simplified this network as an unweighted network by ignoring weight, according to the method in [22].

The network possesses rich metadata which describes the structural and functional roles of each vertex. Therefore, we can evaluate the performance of different methods by measuring how well the detected community reflect the metadata. We choose CPM to be compared in terms of overlapping community detection, and select Louvain method to be compared in terms of disjoint community detection. CPM does not assign all the vertices in a network, and some of them are considered as “background” vertices and belong to no communities. Thus, when compared with CPM, we filter the “background” vertices and only adopt the remaining subnetwork. However, when comparing our method with Louvain method, we use the whole network as our target network. Because the number of communities *K* will also affect the evaluation results, we first set *K* in our model the same as that of each method compared. Specifically, when compared with CPM, we use the number of communities *K* got by CPM in our model. Similarly, when compared with Louvain method, we specify the *K* got by Louvain in our model. Furthermore, we also use the method introduced in “Analysis of the resolution parameter” to automatically determine *K*. To be specific, we got *K* = 958 communities on the subnetwork filtered by CPM to compare with CPM, and we got *K* = 961 communities on the whole network to compare with Louvain method.

We use the WordNet database for the metadata [45], which is specifically built for semantic analysis. This database assignes a set of meanings/definitions to each word, known as Synsets. Moreover, a unique ID is also assigned to each detailed meaning of a word. This enables us to make quantitative analysis. In principle, a pair of words can be considered to be similar if they belongs to a same Synset. We can measure the quality of detected community structure by the enrichment of vertex pair similarity [22]. According to [22], the enrichment of vertex pair similarity is

$$\begin{array}{c} Enrichment=\frac{{\left\langle \mu \left(i,j\right)\right\rangle }_{all\;i,\;j\;within\;same\;community}}{{\left\langle \mu \left(i,j\right)\right\rangle }_{all\;pairs\;i,\;j}}, \end{array}$$ (25)

where *μ*(*i*, *j*) = 1, if words *i* and *j* belong to the same Synset, or 0, otherwise. In other words, the enrichment is the average metadata similarity between all pairs of vertices that share a community, divided by the average metadata similarity between all pairs of vertices. The denominator serves as a baseline similarity and the larger the enrichment, the more similar the vertices, and this indicates better community structure.

When we specify the number of communities *K* same as CPM, the enrichment got by CPM and our method are 30.75 and 56.31, respectively. In spite of using the filtered network which is in favor of CPM, the performance of our result is still better than that of CPM in terms of real semantic. Furthermore, on the whole network, when we specify *K* same as Louvain method, the enrichment of Louvain method is 15.97, and that of our method is 53.32. This result also indicates the superiority of our method from the point of semantic analysis. Besides, when there is no prior information about the number of communities, the enrichment of our method is 63.42 and 80.45 on the filtered network and the whole network, respectively, which still shows the superiority of our method over CPM and Louvain method.

Here we first analyze all the communities in our result overlapped at a popular word “*DAY*” when we set *K* the same as CPM, which is shown in Table 5. As we can see, our method provides much more semantic information than CPM. The reason is that CPM is a k-clique propagation method, and this is a strong constraint on networks. But in practice, networks in real world do not always consist of cliques, so CPM may loose some useful information. For example, in the first community, we detect “*WEEKEND*”, while CPM does not detect it. Obviously, “*WEEKEND*” is very similar with other words in this community from the perspective of semantic. Therefore, we can say, our approach is free from the clique constraint, and provides more comprehensive semantic information than CPM. Furthermore, we also analyze the communities associated with “*DAY*” when the number of communities *K* is determined automatically. As shown in Table 6, we got the similar community results as that of CPM. In summary, under both these two situations, our method can find significant communities with similar semantic.

**Table 5:** The communities associated with the word *DAY* with *K* got by CPM.

Our result	CPM
FRIDAY, MONDAY, TUESDAY, WEDNESDAY, WEEK, WORK, WEEKEND	FRIDAY, MONDAY, TUESDAY, WEDNESDAY, WEEK, WORK
CLOUDY, FOGGY, RAINY, SUNNY, UNCLEAR	FOGGY, RAINY, SUNNY, CLOUDY
AFTERNOON, EVENING, MORNING, NIGHT, NOON, LUNCH, SUNRISE, OATMEAL	AFTERNOON, EVENING, MORNING, NIGHT, NOON,
DATE, MONTH, TIME, YEAR, CALENDAR, SCHEDULE, ALMANAC, COUPLE	DATE, MONTH, TIME, YEAR, CALENDAR

**Table 6:** The communities associated with the word *DAY* with *K* determined automatically.

Our result	CPM
FRIDAY, MONDAY, TUESDAY, WEDNESDAY, WEEK, WEEKEND	FRIDAY, MONDAY, TUESDAY, WEDNESDAY, WEEK, WORK
FOGGY, RAINY, SUNNY, CLOUDY	FOGGY, RAINY, SUNNY, CLOUDY
AFTERNOON, EVENING, MORNING, NOON, SUNRISE	AFTERNOON, EVENING, MORNING, NIGHT, NOON,
DATE, MONTH, YEAR, CALENDAR	DATE, MONTH, TIME, YEAR, CALENDAR

## Discussion

In this work, we propose a novel generative model to detect overlapping and hierarchical community structures, which is based on nonnegative matrix factorization with *l*_2,1_ norm regularization term, balanced by a resolution parameter. In this approach, the NMF technology provides the overlapping communities solution, and the *l*_2,1_ norm regularization term enables us to solve the problem of model selection, i.e., to learn the number of communities automatically. Besides, by varying the resolution parameter, we get the hierarchical organization of networks so as to reveal more comprehensive information. All of these above problems are essential to be solved when dealing with the real-world applications, and here we provide a unified framework. Furthermore, we derive the update rule of the model parameters and give the proof of its correctness. In addition, because our approach is based on NMF, it can not only capture the membership of a vertex in multiple communities, but also measure how strongly that a vertex participates in each of the communities. Finally, the experiments on both of synthetic and real-world networks are presented to show the effectiveness of our approach.

However, our method is not perfect, which still has room for further improvements. Our generative model is originally designed for static network. But in many cases, the static network can be considered as a snapshot in the process of network evolution. Therefore, we wish to extend our approach to detect communities as well as its evolution in the dynamic situations. A natural extension maybe design a effective regularization term that smoothes the current membership matrix and the membership matrix in the next time stamp. This will be our main direction in the future.

## References

[1] Watts DJ, Strogatz SH. Collective dynamics of ‘small-world’ networks. Nature. 1998;393:440–442. 10.1038/30918 9623998

[2] Barabási AL, Albert R. Emergence of scaling in random networks. science. 1999;286(5439):509–512. 10.1126/science.286.5439.509 10521342

[3] Girvan M, Newman MEJ. Community structure in social and biological networks. Proceedings of the National Academy of Sciences. 2002;99(12):7821–7826. 10.1073/pnas.122653799 PMC122977 12060727

[4] Palla G, Derényi I, Farkas I, Vicsek T. Uncovering the overlapping community structure of complex networks in nature and society. Nature. 2005;435(7043):814–818. 10.1038/nature03607 15944704

[5] Fortunato S. Community detection in graphs. Physics Reports. 2010;486(3):75–174. 10.1016/j.physrep.2009.11.002

[6] Xie J, Kelley S, Szymanski BK. Overlapping community detection in networks: the state of the art and comparative study. arXiv preprint arXiv:11105813. 2011;.

[7] Newman M. Communities, modules and large-scale structure in networks. Nature Physics. 2012;8(1):25–31. 10.1038/nphys2162

[8] Karrer B, Newman ME. Stochastic blockmodels and community structure in networks. Physical Review E. 2011;83(1):016107 10.1103/PhysRevE.83.016107 21405744

[9] Zarei M, Izadi D, Samani KA. Detecting overlapping community structure of networks based on vertex–vertex correlations. Journal of Statistical Mechanics: Theory and Experiment. 2009;2009(11):P11013 10.1088/1742-5468/2009/11/P11013

[10] Psorakis I, Roberts S, Ebden M, Sheldon B. Overlapping community detection using Bayesian non-negative matrix factorization. Physical Review E. 2011;83(6):066114 10.1103/PhysRevE.83.066114 21797448

[11] Wang F, Li T, Wang X, Zhu S, Ding C. Community discovery using nonnegative matrix factorization. Data Mining and Knowledge Discovery. 2011;22(3):493–521. 10.1007/s10618-010-0181-y

[12] Zhang Y, Yeung DY. Overlapping community detection via bounded nonnegative matrix tri-factorization. In: Proceedings of the 18th ACM SIGKDD international conference on Knowledge discovery and data mining. ACM; 2012. p. 606–614.

[13] Zhang ZY, Wang Y, Ahn YY. Overlapping community detection in complex networks using symmetric binary matrix factorization. Physical Review E. 2013;87(6):062803 10.1103/PhysRevE.87.062803 23848725

[14] Cao X, Wang X, Jin D, Cao Y, He D. Identifying overlapping communities as well as hubs and outliers via nonnegative matrix factorization. Scientific reports. 2013;3,2993 10.1038/srep02993 24129402 PMC3797436

[15] Ren W, Yan G, Liao X, Xiao L. Simple probabilistic algorithm for detecting community structure. Physical Review E. 2009;79(3):036111 10.1103/PhysRevE.79.036111 19392022

[16] Shen HW, Cheng XQ, Guo JF. Exploring the structural regularities in networks. Physical Review E. 2011;84(5):056111 10.1103/PhysRevE.84.056111 22181477

[17] Brunet JP, Tamayo P, Golub TR, Mesirov JP. Metagenes and molecular pattern discovery using matrix factorization. Proceedings of the National Academy of Sciences. 2004;101(12):4164–4169. 10.1073/pnas.0308531101 PMC384712 15016911

[18] Tan VY, Fevotte C. Automatic Relevance Determination in Nonnegative Matrix Factorization with the/spl beta/-Divergence. Pattern Analysis and Machine Intelligence, IEEE Transactions on. 2013;35(7):1592–1605. 10.1109/TPAMI.2012.240 23681989

[19] Lancichinetti A, Fortunato S, Kertész J. Detecting the overlapping and hierarchical community structure in complex networks. New Journal of Physics. 2009;11(3):033015 10.1088/1367-2630/11/3/033015

[20] Blondel VD, Guillaume JL, Lambiotte R, Lefebvre E. Fast unfolding of communities in large networks. Journal of Statistical Mechanics: Theory and Experiment. 2008;2008(10):P10008 10.1088/1742-5468/2008/10/P10008

[21] Huang J, Sun H, Liu Y, Song Q, Weninger T. Towards online multiresolution community detection in large-scale networks. PloS one. 2011;6(8):e23829 10.1371/journal.pone.0023829 21887325 PMC3161084

[22] Ahn YY, Bagrow JP, Lehmann S. Link communities reveal multiscale complexity in networks. Nature. 2010;466(7307):761–764. 20562860 10.1038/nature09182

[23] Lee H, Choi S. Group nonnegative matrix factorization for EEG classification. In: International Conference on Artificial Intelligence and Statistics; 2009. p. 320–327.

[24] Kim J, Monteiro R, Park H. Group Sparsity in Nonnegative Matrix Factorization In: SDM. SIAM; 2012 p. 851–862.

[25] Lee DD, Seung HS. Learning the parts of objects by non-negative matrix factorization. Nature. 1999;401(6755):788–791. 10.1038/44565 10548103

[26] Oja E. Principal components, minor components, and linear neural networks. Neural Networks. 1992;5(6):927–935. 10.1016/S0893-6080(05)80089-9

[27] Jin D, He D, Hu Q, Baquero C, Yang B. Extending a configuration model to find communities in complex networks. Journal of Statistical Mechanics: Theory and Experiment. 2013;2013(09):P09013 10.1088/1742-5468/2013/09/P09013

[28] Boyd SP, Vandenberghe L. Convex optimization. Cambridge university press; 2004.

[29] Rosvall M, Bergstrom CT. Maps of random walks on complex networks reveal community structure. Proceedings of the National Academy of Sciences. 2008;105(4):1118–1123. 10.1073/pnas.0706851105 PMC2234100 18216267

[30] Esquivel AV, Rosvall M. Compression of flow can reveal overlapping-module organization in networks. Physical Review X. 2011;1(2):021025 10.1103/PhysRevX.1.021025

[31] Danon L, Diaz-Guilera A, Duch J, Arenas A. Comparing community structure identification. Journal of Statistical Mechanics: Theory and Experiment. 2005;2005(09):P09008 10.1088/1742-5468/2005/09/P09008

[32] Newman ME, Girvan M. Finding and evaluating community structure in networks. Physical review E. 2004;69(2):026113 10.1103/PhysRevE.69.026113 14995526

[33] Lancichinetti A, Fortunato S, Radicchi F. Benchmark graphs for testing community detection algorithms. Physical Review E. 2008;78(4):046110 10.1103/PhysRevE.78.046110 18999496

[34] Rosvall M, Bergstrom CT. Multilevel compression of random walks on networks reveals hierarchical organization in large integrated systems. PloS one. 2011;6(4):e18209 10.1371/journal.pone.0018209 21494658 PMC3072965

[35] Fortunato S, Barthelemy M. Resolution limit in community detection.Proceedings of the National Academy of Sciences. 2007;104(1):36–41. 10.1073/pnas.0605965104 PMC1765466 17190818

[36] Zachary WW. An information flow model for conflict and fission in small groups. Journal of anthropological research. 1977;p. 452–473.

[37] Lusseau D, Newman MEJ. Identifying the role that animals play in their social networks. Proceedings of the Royal Society of London Series B: Biological Sciences. 2004;271(Suppl 6):S477–S481. 10.1098/rsbl.2004.0225 15801609 PMC1810112

[38] Newman ME. Modularity and community structure in networks. Proceedings of the National Academy of Sciences. 2006;103(23):8577–8582. 10.1073/pnas.0601602103 PMC1482622 16723398

[39] Newman ME. Finding community structure in networks using the eigenvectors of matrices. Physical Review E. 2006;74(3):036104 10.1103/PhysRevE.74.036104 17025705

[40] Knuth DE. The Stanford GraphBase: a platform for combinatorial computing. AcM Press; 1993.

[41] Duch J, Arenas A. Community detection in complex networks using extremal optimization. Physical review E. 2005;72(2):027104 10.1103/PhysRevE.72.027104 16196754

[42] Lambiotte, R, Delvenne, JC, Barahona, M. Laplacian dynamics and multiscale modular structure in networks. arXiv preprint arXiv:08121770. 2008;.

[43] Lambiotte, R. Multi-scale modularity in complex networks. In: Modeling and optimization in mobile, ad hoc and wireless networks (WiOpt), 2010 Proceedings of the 8th International Symposium on. IEEE; 2010. p. 546–553.

[44] Delvenne JC, Yaliraki SN, Barahona M. Stability of graph communities across time scales. Proceedings of the National Academy of Sciences. 2010;107(29):12755–12760. 10.1073/pnas.0903215107 PMC2919907 20615936

[45] Fellbaum C. WordNet. Springer; 2010.

